# Multifactorial Thresholds of Psychomotor Fatigue in Soccer: A Systematic Review and Meta-Analysis of Randomized Controlled Trials

**DOI:** 10.5114/jhk/212912

**Published:** 2026-04-02

**Authors:** Moses Gnanasigamani, Jennifer M. Kiruba, Paweł Chmura

**Affiliations:** 1 Department of Team Games, Wroclaw University of Health and Sport Sciences, Wrocław, Poland.; 2 Department of Chemical Engineering, Faculty of Chemistry, Wrocław University of Science and Technology, Wroclaw, Poland.; 3 Department of Sports Science and Clinical Biomechanics, Sport and Health Sciences Cluster (SHSC), University of Southern Denmark, Odense, Denmark.

**Keywords:** fatigue interventions, multimodal fatigue, recovery strategies, technical performance

## Abstract

The present study addressed the complex nature of fatigue in soccer, examining its physical, psychological, neuromuscular, and metabolic dimensions. It evaluated the impact of these different types of fatigue on players’ performance, highlighting the importance of comprehensive fatigue-management strategies for enhancing performance and reducing injury risk among soccer players. The primary aim of this study was to investigate the effects of various types of fatigue on performance of male soccer players across different competitive levels, through a systematic review and meta-analysis of randomized controlled trials. A total of 37 randomized controlled trials involving male soccer players were included, following the PRISMA (Preferred Reporting Items for Systematic Reviews and Meta-Analyses) guidelines to assess the multifaceted impacts of fatigue. Key findings revealed that neuromuscular fatigue had the highest mean effect size (0.63), with substantial consistency across studies (95% CI: 0.45–0.80, I^2^ = 99.79%). Physical and metabolic fatigue both showed a mean effect size of 0.38, though they differed in variability; metabolic fatigue demonstrated considerable heterogeneity (I^2^ = 98.73%), reflecting diverse physiological responses, while physical fatigue showed moderate consistency (I^2^ = 98.20%). Psychological fatigue had a significant impact on performance (mean effect size: 0.57), with variability (I^2^ = 97.08%) suggesting context-dependent effects. These results underscore the necessity of a multimodal approach that integrates physical, metabolic, neuromuscular, and psychological interventions to optimize soccer performance and mitigate injury risk. Practical implications include the adoption of targeted recovery strategies such as inter-set recovery intervals and whole-body vibration techniques, as well as the implementation of mental resilience and cognitive training to manage psychological fatigue. Such strategies are essential for developing individualized training and recovery protocols that enhance athletic performance and support long-term career sustainability.

## Introduction

During the past few decades, soccer has undergone significant transformations, marked by noticeable increases in game intensity, players’ performance demands, and overall pace (Clemente et al., 2022). Modern soccer requires players to operate at a faster pace while running freely and with the ball (Caldbeck and Dos Santos, 2022). This evolution requires advanced players’ training, fatigue management, and strategic adaptations to maintain optimal performance. The dynamic nature of soccer, where tactical and physical demands uniquely interact in each match, requires a rigorous analysis of how various forms of fatigue interact and affect decision making, skill execution, and injury risk (Jastrzębski et al., 2025). Effective performance in soccer depends not only on physical endurance, but also on cognitive sharpness and the ability to adapt to rapidly changing scenarios during matches.

Moreover, the escalation in the number of domestic league matches, international club competitions such as the UEFA Champions League, the Europa League, and other international fixtures has led to congested schedules (Chmura et al., 2019). Shortened off-seasons exacerbate this issue, leaving players with insufficient time to fully recover, which heightens the risk of cumulative fatigue and injuries. Understanding the factors contributing to fatigue is therefore critical to maintaining players’ health and performance throughout demanding schedules (Djaoui et al., 2022; Pillay et al., 2022). Game duration frequently exceeds 90 min due to stoppages and extra time, requiring players to sustain peak physical and mental performance, especially in high-stakes matches (Suarez-Arrones et al., 2020). Effective fatigue management is crucial to ensuring optimal performance throughout the duration of matches (Lourenço et al., 2023). This shift requires specialised training regimes that focus on skill execution under fatigue and high-intensity conditions, coupled with rapid adaptation to dynamic game situations (Unnithan et al., 2012). Consequently, cognitive training and mental resilience have become integral components of modern training programmes in the last few seasons (Konter et al., 2019). High-Intensity Interval Training (HIIT) has been widely adopted to improve anaerobic capacity and the ability to perform repeated high-intensity efforts, reflecting the increased physical and cognitive demands of the sport (Costello et al., 2022; Mohr et al., 2003; Spieszny et al., 2022). HIIT and high-volume running training (HVT) have been effective in improving endurance performance in youth soccer players. Faude et al. (2013) showed that increases in high-intensity sprints and distances travelled at high speeds required players to reach optimal physical fitness through improved endurance, strength, and agility to alter the score or successfully score a goal.

Understanding the multifaceted nature of fatigue in soccer is imperative to optimise players’ performance and health. Soccer, characterised by its high intensity and long-duration effort, imposes significant psycho-physiological, neuromuscular, and metabolic challenges on athletes. As the global popularity of soccer increases, players' demands also increase, underscoring the need for effective fatigue management strategies (Varjan et al., 2024). Despite extensive research on various aspects of fatigue, inconsistencies and knowledge gaps persist, highlighting the need for a comprehensive synthesis through systematic review and meta- analysis of the literature.

Fatigue in soccer affects performance of players, increasing the risk of injury and affecting physical, psychological, neuromuscular, and metabolic functions. Understanding fatigue is crucial to developing conditioning programmes that would improve performance and recovery, as well as would contribute to injury prevention (Silva et al., 2018). Mental fatigue significantly affects physical, technical and tactical performance, influencing decision making and players’ synchronisation (Kunrath et al., 2020). Monitoring fatigue through objective and subjective tests provides a comprehensive understanding, which aids in effective training and recovery management (Lourenço et al., 2023). Furthermore, fatigue analysis is essential for preventing injuries, particularly during high-intensity match play (de Oliveira et al., 2019; Mohd Faozi and Raja Azidin, 2023).

**Figure 1:**
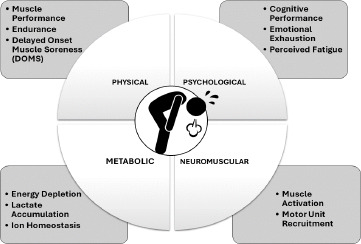
Factors affecting different types of fatigue (physical, metabolic, neuromuscular, psychological) and their characteristics.

Fatigue in soccer encompasses various types, each with its unique impact on performance. Physical fatigue arises from prolonged activity, leading to decreased muscle performance, reduced sprint speed, and increased muscle soreness, affecting neuromuscular functions and requiring significant recovery time (Rampinini et al., 2011). Muscle fatigue impacts mechanical movements, reducing the speed and accuracy of ball delivery, especially when combined with mental fatigue, leading to significant performance impairment (Díaz-García, 2021). Match-related fatigue appears during specific match periods, linked to muscle ion homeostasis disturbances and low glycogen levels (Mohr et al., 2010). Perceived fatigue affects both general and specific aspects of performance, with task motivation mitigating these effects (Barte et al., 2017). Mental fatigue alters psycho-physiological responses, cognitive performance, and technical skills, reducing overall effectiveness in the field, often due to high cognitive demands such as decision making and maintaining attention (Lourenço et al., 2023; Soylu et al., 2022) (Figure 1). Effective players’ rotation and strategic substitutions can help manage cumulative fatigue over a season. Veugelers et al. (2016) demonstrated that frequent submaximal tests can help monitor and manage players' physiological states without inducing additional fatigue, thus optimising their performance during matches.

This systematic review and meta-analysis aimed to strengthen the scientific understanding underpinning modern soccer by synthesizing diverse findings on the physiological, psychological, neuromuscular, and metabolic aspects of fatigue. The results provide evidence-based insights into the mechanisms and impacts of fatigue, highlighting effective interventions such as high-intensity interval training, nutritional strategies, and recovery protocols.

## Methods

### Study Design and Protocol

This systematic literature review adhered to the Preferred Reporting Items for Systematic Reviews and Meta-Analyses (PRISMA) guidelines (Page et al., 2021). The primary objective was to compile and critically evaluate research on the analysis of fatigue in soccer players, considering various dimensions of fatigue, including physical, psychological, neuromuscular and metabolic aspects.

### Data Sources and Search Strategy

Research articles were systematically retrieved from three major electronic databases: Scopus, Web of Science (WoS), and PubMed. Keywords and Boolean operators were tailored to each database to retrieve studies relevant to physical, psychological, neuromuscular, and metabolic fatigue. To identify studies on physical fatigue, the search included terms such as “physical”, “VO_2_”, “sprint”, “HRV” (heart rate variability), and “countermovement”. Studies addressing psychological fatigue were retrieved using terms like “mental”, “cognitive”, “psycho*” (addition of asterisks indicates that the keyword was truncated to include its variations), “psychomotor”, “perceived” and “reaction”. Keywords for neuromuscular fatigue included “neuromuscl*”, “peripheral”, “central”, “force” and “electromyo*”. To explore metabolic fatigue, the search terms incorporated “blood lactate”, “glycogen”, “blood pH”, “oxygen”, “respirat” (to include respiration and respiratory), “ammoni” (to include ammonia), “aerobic” and “anaerobic”. These terms were combined with “fatigue” and the sport-specific terms “soccer” OR “football” to focus on soccer-related contexts. The search targeted studies assessing fatigue in soccer players published from 2013 to 2023, restricting inclusion to articles written in English and published in peer-reviewed journals. The type of the study was specifically limited to randomised controlled trials (RCTs) to ensure the retrieval of high-quality empirical research.

### Eligibility Criteria

Eligibility was defined using the PICOS (Population, Intervention, Comparison, Outcome, Study Design) framework, which facilitated a structured and systematic approach to define the research objective (Cooke et al., 2012) (Table 1). The framework included various performance levels of players (youth, amateur, professional, and elite) and considered different fatigue assessments and interventions. Inclusion criteria were established to include studies that involved soccer players of all performance levels, employing validated metrics for fatigue assessment such as VO_2max_ tests, lactate thresholds and GPS tracking, among others. Exclusion criteria were applied to non-English publications, non-RCTs, and studies focussing on other sports or conditions not directly related to soccer (Table 2).

**Table 1:** PICOS framework for establishing the research objective.

Element	Details
**Population (P)**	Soccer players at different levels: youth, amateur, professional, elite; including subgroups by age, gender, professional level, and playing position.
**Intervention (I)**	Fatigue-related assessments and interventions addressing different types of fatigue based on study measures:
	- Metabolic: Interventions like curcumin supplementation for muscle function recovery.
	- Physical: Strategies like hydration/dousing for physiological regulation (e.g., rectal temperature, heart rate).
	- Neuromuscular: Training regimens affecting the RSI or CMJ performance.
	- Psychological: Interventions or cognitive-behavioral training to reduce stress and improve mental recovery.
**Comparator (C)**	Comparisons include:
	- Control vs. intervention groups (e.g., placebo vs. curcumin supplementation).
	- Pre- and post-intervention measurements within the same players to assess fatigue-related recovery.
	- Subgroup comparisons, such as players at different levels of play, age, or playing positions.
**Outcome (O)**	Outcomes are categorized based on fatigue types:
	- Physical: Muscle function recovery measured via CMJ height or the RSI.
	- Metabolic: Biomarkers like CK levels, hydration status, heart rate, or rectal temperature.
	- Neuromuscular: Reactive strength and muscle coordination.
	- Psychological: Subjective or cognitive measures (though not detailed in this dataset). Measurement tools include HR monitors, biomarkers, and performance metrics.
**Study Design (S)**	Randomized controlled trials or observational studies analyzing fatigue outcomes. Focus is on interventions and their impact on specific fatigue markers, using high-quality methods with sufficient sample sizes and rigorous control.

**Table 2:** Inclusion and exclusion criteria set for the preliminary screening of the retrieved articles.

Criteria Type	Category	Inclusion Criteria	Exclusion Criteria
**Participants**	Study Population	Studies involving soccer players at all levels: youth, professional, elite.	Studies not involving soccer players or focusing exclusively on other sports. Studies focusing on populations with specific health conditions or disabilities that may independently affect fatigue (unless the condition is a direct result of soccer play).
**Study Type**	Publication Type	Only original research articles that performed randomized controlled trial (RCT) studies were considered	Reviews, editorials, opinion pieces, theoretical papers, conference abstracts, letters, and book chapters.
**Outcomes**	Measurement Focus	Studies measuring fatigue thresholds using validated metrics for cognitive, physical, psychological, neuromuscular, and functional fatigue.	Studies without specific data on fatigue thresholds or undefined measurement methods.
**Tools**	Measurement Tools	Studies using validated tools such as VO_2max_ tests, lactate thresholds, psychomotor vigilance tasks, heart rate variability, and GPS tracking.	Studies with unclear or non-validated measurement tools.
**Language**	Language of Publication	Articles published in English.	Articles published in languages other than English
**Publication Date**	Time Frame	Studies published within the last 10 years (2013 onwards)	Studies older than 10 years.
**Research Quality**	Quality Assurance	Studies with sufficient methodological details to understand fatigue assessment processes, validated by peer-review (risk of bias assessment).	Studies with insufficient methodological details or lacking peer-review.

### Data Extraction and Management

Data extraction was meticulously performed using Zotero to ensure accurate data capture and management. Retrieved articles were initially exported in the CSV format from the selected databases and were merged and deduplicated to refine the dataset. Each article underwent rigorous evaluation to extract relevant data such as age, gender, the fatigue type, sample size, effect sizes, and study quality. Each entry was categorized into four distinct physiological domains of fatigue indicators—physical, neuromuscular, metabolic, and psychological—and was predicated on a rigorous assessment of dependent and independent variables alongside the specific measures employed in each study.

### Data Synthesis

In synthesizing the data, we categorized studies based on the types of fatigue: physical, metabolic, neuromuscular, and psychological. This classification framework relied on the specific dependent and independent variables used in each study, as well as the measures employed for assessing fatigue outcomes. Studies were extracted systematically, and measures such as countermovement jump (CMJ) height, the reactive strength index (RSI), hydration status, and subjective psychological surveys were mapped to respective fatigue categories through a robust charting process.

The dependent and independent variables were central to this categorization. For example, interventions such as betaine supplementation targeting muscle recovery (independent variable) were paired with outcomes like sprint time (dependent variable) to classify a study under the physical type of fatigue. Similarly, training and competition exposure that were matched with physiological markers such as creatine kinase (CK) levels, were classified as metabolic fatigue. Neuromuscular fatigue was identified through measures like electromyography (EMG) and force, while subjective ratings such as the rating of perceived exertion (RPE), were linked to psychological fatigue studies.

To ensure consistency, a summary of findings table was developed to chart the relationships among interventions, measures, and dependent and independent variables. This table included elements such as measures, dependent and independent variables, effect sizes, confidence intervals, and categorization into one of the four fatigue types.

### Risk of Bias Assessment

The methodological quality assessment of RCT studies was conducted through the Risk of Bias 2 (RoB 2) tool (Crocker et al., 2023; Fernández et al., 2023). This tool assesses bias across five domains: randomization process, deviations from intended interventions, missing outcome data, measurement of the outcome, and selection of the reported result. All the steps of the evaluation process were conducted independently by two reviewers, with discrepancies resolved by a third reviewer. The summary of the findings from the risk of bias assessment is discussed later.

### Statistical Analysis

Effect sizes for each entry were recorded along with 95% confidence intervals. Where confidence intervals were not initially reported but relevant standard deviation or error data were available, confidence intervals were calculated accordingly. Standard error was determined for each study, facilitating an assessment of heterogeneity via the I^2^ Statistic (Higgins et al., 2003). Low heterogeneity was defined as an I^2^ value of less than 25%, indicating minimal variability among study results. Moderate heterogeneity was characterized by an I^2^ value between 25% and 75%, reflecting a moderate level of variability. High heterogeneity was defined as an I^2^ value exceeding 75%, suggesting substantial variability that might affect the generalizability of pooled results. These thresholds were used to evaluate and interpret the consistency of findings across studies. The risk of reporting bias was evaluated using the Egger’s test (Egger et al., 1997), where a *p*-value below 0.05 suggested the presence of potential publication bias. Sensitivity analyses were conducted utilizing the trim and fill method in RStudio (2023.06.0 Build 421) (Shi and Lin, 2019).

Furthermore, a pooled effect size and the corresponding confidence interval were computed for each category, assuming the random-effects model and a normal distribution and adhering to a 95% confidence level. Results were synthesized into a forest plot for each category, generated through custom Python coding to visually represent the effect sizes and their confidence intervals, assuming a normal distribution at a 95% confidence level.

## Results

### Study Selection and Characteristics

**Figure 2:**
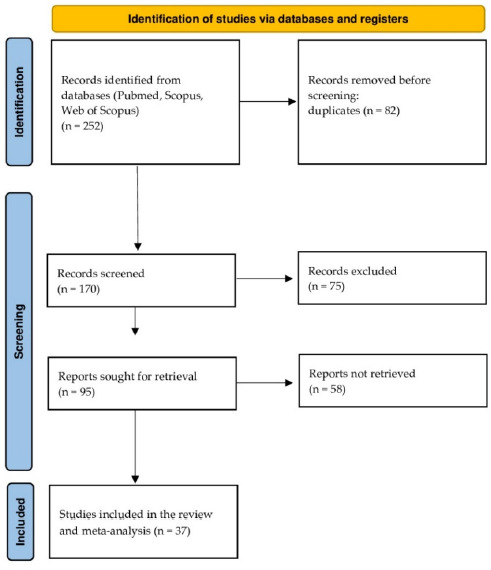
PRISMA methodology flowchart.

The process of choosing relevant studies using the PRISMA methodology is illustrated in Figure 2. In general, 252 studies were identified from multiple databases. They were subjected to multiple screening steps such as elimination of duplicates and inclusion/exclusion criteria. The inaccessibility of relevant data and access to the article led to a final set of studies utilised in this study. Finally, a total of 37 studies were included in the systematic literature review and meta-analysis.

Five of the studies employed a crossover randomised design (Abbott et al., 2023; Annino et al., 2022; Benjamin et al., 2021; Botek et al., 2022; Nakamura et al., 2020), while others followed a parallel group control (Arslan et al., 2021; Asgari et al., 2022; Campbell et al., 2020; Hammami et al., 2020; Huerta Ojeda et al., 2024; Mendez-Rebolledo et al., 2021) and a single-blind randomised trial (Boraczyński et al., 2023). The studies exclusively involved male soccer players at various levels: amateur (Arslan et al., 2021; Campbell et al., 2020), semi-professional (Asgari et al., 2022), and professional (Abbott et al., 2023; Annino et al., 2022; Boraczyński et al., 2023). Sample sizes ranged from ten (Campbell et al., 2020) to 90 participants (Asgari et al., 2022).

### Quality of the Studies

**Figure 3:**
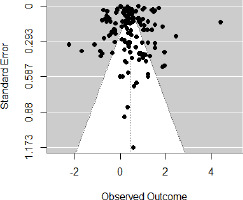
Funnel plot depicting minimal publication bias.

The quality of the included studies was evaluated on the basis of the Risk of Bias (RoB). Most studies showed a low risk of bias, particularly in handling incomplete outcome data and general integrity of the study (Table 3). However, several studies exhibited some concerns about random sequence generation and blinding of participants and personnel, while a few showed moderate risk in these areas and selective reporting. Only one study showed high risk in blinding the outcome assessment, and none fell into the critical risk category. Studies such as Annino et al. (2022) and Hammami et al. (2020) demonstrated high methodological quality, while Boraczyński et al. (2023) and Smith et al. (2016) presented moderate risks. Minimal publication bias was determined by the trim and fill method (Figure 3).

**Table 3:** Results of a methodological quality assessment of included studies using the RoB 2 tool.

Study	D1a	D1b	D2	D3	D4	D5	DS	Overall
Abbott et al. (2024)	SC	–	SC	L	L	SC	–	SC
Annino et al. (2022)	L	L	L	L	L	L	L	L
Arslan et al. (2021)	L	L	L	SC	L	SC	SC	L
Asgari et al. (2022)	L	L	M	L	L	L	L	L
Barte et al. (2019)	SC	–	SC	L	L	SC	L	SC
Benjamin et al. (2021)	SC	–	SC	L	L	SC	L	SC
Boraczyński et al. (2023)	L	NI	M	L	NI	M	M	L
Botek et al. (2022)	SC	–	SC	L	L	SC	L	SC
Campbell et al. (2020)	L	SC	L	L	NI	L	L	L
Casamichana et al. (2022)	M	M	L	L	M	L	L	L
Castilla-López and Romero-Franco (2023)	L	L	L	L	L	L	NI	L
Dello Iacono et al. (2019)	SC	–	SC	L	L	SC	L	SC
Fernandes et al. (2021)	L	SC	–	–	NI	L	L	L
Ferreira et al. (2024)	SC	–	SC	SC	L	L	–	SC
Gallo et al. (2017)	L	NI	L	L	M	L	L	L
Guerrero et al. (2019)	SC	–	SC	SC	L	L	–	SC
Hammami et al. (2016)	L	SC	L	L	NI	L	L	L
Hammami et al. (2020)	L	L	L	L	L	L	L	L
Hammami et al. (2022)	L	NI	L	L	L	M	L	L
Huerta Ojeda et al. (2024)	L	SC	L	L	L	L	–	L
Keller et al. (2024)	L	–	L	L	L	L	–	L
Kritikos et al. (2021)	L	SC	L	L	NI	L	L	L
Kryściak et al. (2023)	L	NI	L	M	L	M	L	M
Mendez-Rebolledo et al. (2021)	SC	–	SC	L	L	SC	L	SC
Moran et al. (2022)	SC	–	SC	L	L	SC	L	SC
Nakamura et al. (2020)	SC	–	SC	L	L	SC	L	SC
Nédélec et al. (2012)	L	NI	L	L	L	L	L	L
Nobari et al. (2021)	L	SC	L	L	SC	L	L	L
Pareja-Blanco et al. (2016)	SC	–	SC	H	L	SC	L	H
Pinheiro et al. (2024)	L	–	SC	SC	L	L	–	L
Rabbani et al. (2019)	L	SC	–	–	NI	L	L	L
Ramirez-Campillo et al. (2019)	L	SC	L	L	NI	L	L	L
Ripley et al. (2023)	SC	SC	SC	H	L	SC	L	SC
Ruddy et al. (2020)	L	SC	–	–	L	L	L	L
Smith et al. (2016)	L	L	M	M	M	L	NI	M
Veugelers et al. (2016)	M	L	M	L	L	M	L	M
Zhou et al. (2022)	SC	–	SC	L	L	SC	L	SC

Note: D1a: Random Sequence Generation; D1b: Allocation Concealment; D2: Blinding of Participants and Personnel; D3: Blinding of Outcome Assessment; D4: Incomplete Outcome Data; D5: Selective Reporting; DS: Other Bias. C: Critical; H: High risk; L: Low risk; M: Moderate; NI: No information; S: Serious; SC: Some concerns

### Comparison of Fatigue Outcomes

**Figure 4:**
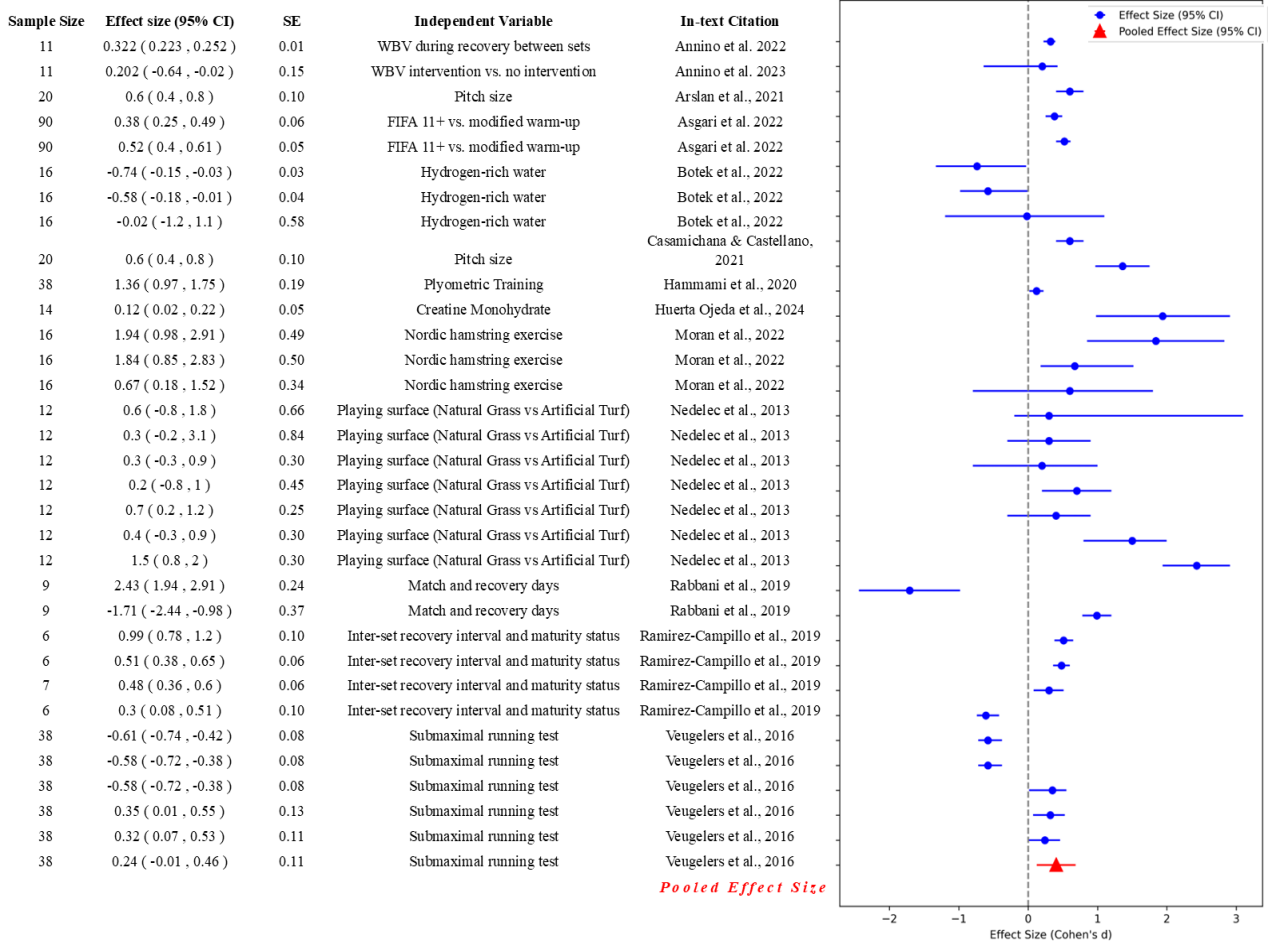
The forest plot for various physical measures for fatigue in soccer. This forest plot depicts the effect sizes (Cohen's d) for various physical measures related to fatigue, as referenced by the respective studies. Each blue circle represents the effect size for an individual study, with horizontal lines indicating the 95% confidence intervals. The red triangle at the bottom represents the pooled effect size, calculated as the mean effect size across all studies, with its corresponding 95% confidence interval. The vertical grey dashed line indicates the reference point of no effect (effect size of 0). The y-axis lists the studies by their in-text citations, with the pooled effect size shown at the bottom

**Figure 5:**
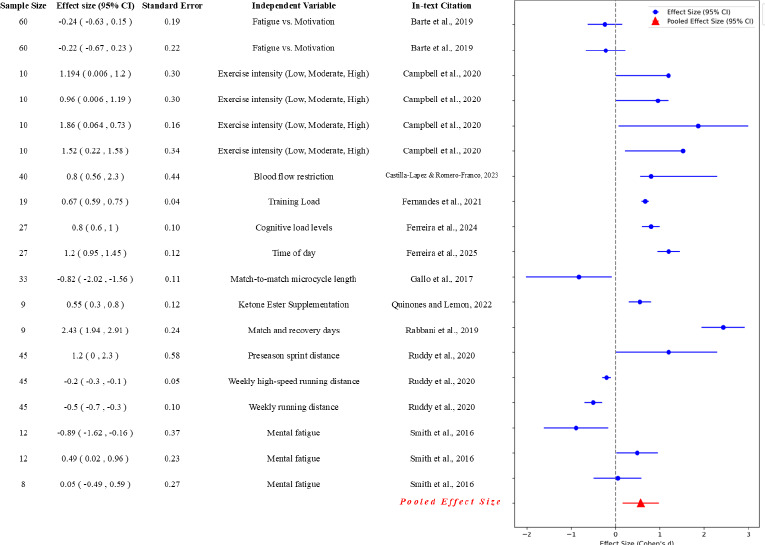
The forest plot for various psychological measures for fatigue in soccer.

**Figure 6:**
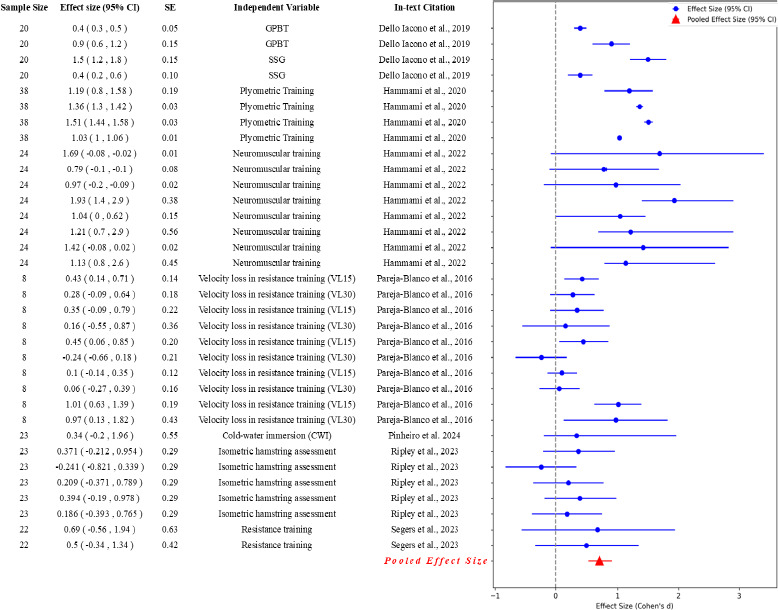
The forest plot for various neuromuscular measures for fatigue in soccer.

**Figure 7:**
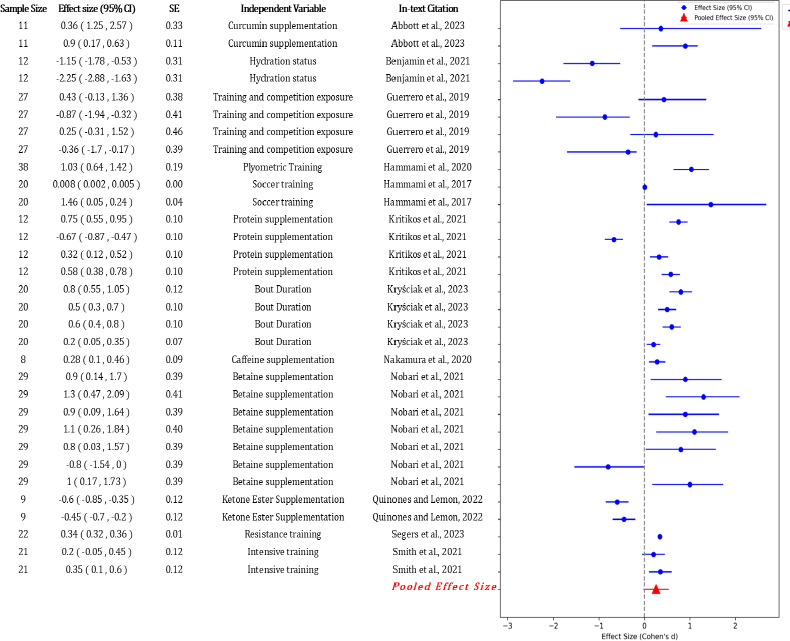
The forest plot for various metabolic measures for fatigue in soccer.

The results of the meta-analysis for each type of fatigue are presented in Figures 4–7. The meta-analysis in multiple studies investigating the effects of physical activity interventions on fatigue outcomes included various performance measures. Studies assessed the various dimensions of fatigue through multiple measures, such as heart rate variability, agility tests, and physical demand indices, among others.

In the category of physical fatigue, the Q statistic was considerably lower at 2,134.92 with 39 degrees of freedom, but still indicated significant heterogeneity (I^2^ of 98.17%). The size of the pooled effect was 0.30, which was less than that observed in neuromuscular fatigue studies, with a standard error of 0.06 and a confidence interval of 0.18 to 0.41. This suggests that physical fatigue, although substantial, had a slightly less pronounced effect compared to neuromuscular fatigue.

Psychological fatigue had the smallest sample heterogeneity metrics, with a Q statistic of 616.25 and 18 degrees of freedom. However, the I^2^ statistic remained very high at 97.08%, indicating that variability was still predominantly due to genuine differences among studies. The pooled effect size of 0.55 was the highest among all categories, with a notably wide confidence interval ranging from 0.21 to 0.89 and the largest standard error of 0.17. This implies that psychological fatigue could vary considerably among soccer players and might have had a substantial impact.

For neuromuscular fatigue, a Q statistic (23,246.30) with 48 degrees of freedom indicated a significant amount of heterogeneity among the included studies. This was further confirmed by a high I^2^ statistic of 99.79%, suggesting that nearly all of the variability in effect size was due to real differences in the study results rather than sampling error. The size of the pooled effect, a measure of the average magnitude of neuromuscular fatigue, was 0.48 with a standard error of 0.07. The confidence interval ranged from 0.33 to 0.63, implying a moderate to high effect of neuromuscular fatigue in soccer players.

Lastly, metabolic fatigue showed a Q statistic of 2,436.51 with 31 degrees of freedom and an I^2^ statistic of 98.73%, indicating substantial heterogeneity among the studies analysed. The size of the pooled effect was 0.28, similar to physical fatigue, with a confidence interval of 0.14 to 0.42. The standard error was slightly higher at 0.07, reflecting slightly greater variability in the effect sizes among studies in this category.

When comparing the pooled effect sizes, psychological fatigue stood out with the highest effect size (0.55), indicating that interventions had a more substantial impact on reducing psychological fatigue. This suggests that mental skill training and stress management strategies were effective in improving psychological well-being in athletes. Physical fatigue had a small effect size (0.3), showing that interventions moderately reduced physical fatigue through active recovery, nutrition, and periodized training programs. Neuromuscular fatigue had a larger effect size (0.48), reflecting modest improvements through neuromuscular training, although these effects varied widely. Metabolic fatigue had the least pooled effect size (0.28), implying minimal impact from interventions, possibly due to complex metabolic processes influenced by various factors in soccer.

The study reveals significant insights into the physiological and performance-related impact of various interventions. Abbott et al. (2023) demonstrated that curcumin supplementation significantly improved muscle function, as evidenced by increased countermovement jump height (CMJ) and the reactive strength index (RSI) with effect sizes of 0.36 and 0.90, respectively. These findings underscore curcumin's potential as a beneficial supplement for post-exercise recovery and performance enhancement.

Benjamin et al. (2021) investigated physiological effects of hydration and dousing, showing substantial decreases in the heart rate and rectal temperature, with effect sizes of −1.15 and −2.25. These interventions are crucial for physiological recovery, maintaining performance, and preventing overheating during intense physical activities. Guerrero et al. (2019) assessed creatine kinase (CK) levels in 27 participants, highlighting muscle damage and adaptation during the pre-season with an effect size of 0.43. This study emphasized the importance of monitoring muscle damage and implementing appropriate recovery strategies throughout training cycles. Furthermore, Annino et al. (2022) explored blood lactate concentration and sprint time, finding effect sizes of 0.32 and 0.20 for faster recovery and performance maintenance using whole-body vibration (WBV) during recovery. Arslan et al. (2021) reported a moderate increase in players’ load with varying pitch sizes, with an effect size of 0.6. Asgari et al. (2022) examined agility and dribbling performance in 90 players, demonstrating medium to large improvements with the FIFA 11+ warm-up routine, with effect sizes of 0.38 and 0.52, respectively.

Studies revealed significant decreases in passing accuracy, fatigue levels, and general wellness, with effect sizes ranging from −0.24 to 1.86, underscoring the holistic impact of mental fatigue. Barte et al. (2019) and Campbell et al. (2020) highlighted the importance of mental resilience and motivation in mitigating the negative effects of mental fatigue.

In terms of neuromuscular fatigue, heavy-resistance training showed substantial improvements in sprint times and jump heights, with Boraczyński et al. (2023) reporting decreases in 10-m and 30-m sprint times and increases in CMJ and squat jump heights. Dello Iacono et al. (2019) confirmed the efficacy of Game Profile-Based Training (GPBT) in enhancing sprint performance.

## Discussion

### Physical Fatigue

Physical fatigue in soccer players often results from high intensity activities such as sprinting, jumping, and continuous running, leading to the depletion of energy stores and the accumulation of metabolic by-products (Jaspers et al., 2017). This type of fatigue has a mean effect size of 0.30, with a confidence interval from 0.18 to 0.42, indicating moderate consistency across studies. Effective interventions include inter-set recovery intervals, match and recovery day strategies, and whole-body vibration techniques, which generally exhibit positive effects. Cloak et al. (2016) demonstrated a consistent positive impact of the inter-set recovery intervention with a mean effect size of 0.36 across ten studies. Physical fatigue's variability in effects was further illustrated by an I^2^ of 98.17%, representing very high heterogeneity, reflecting significant variability in how physical fatigue affected soccer players, though the overall effects were reasonably consistent (Mohr et al., 2003). Studies indicated that fatigue could lead to a significant decrease in sprint performance and agility as games progressed, impacting players' ability to perform high-intensity activities (Dambroz et al., 2022). The combination of physical and mental fatigue exacerbated the negative effects on soccer performance compared to either type of fatigue alone. Additionally, negative effects of physical fatigue were observed on technical fundamentals such as passing, dribbling, and shooting (Díaz-García et al., 2021). Effective warm-up routines, such as the FIFA 11+ programme, enhanced physical performance and reduced injury risk by preparing players physiologically and psychologically for the demands of the game (Pereira et al., 2022; Skalski et al., 2024). The tactical dimension of soccer performance has received less attention in the literature. More research is needed to understand the impact of physical fatigue on the tactical decision-making and positioning of soccer players.

### Metabolic Fatigue

Metabolic fatigue in soccer presented with a mean effect size of 0.28, bounded by a wide confidence interval from 0.14 to 0.42, which was reflective of considerable variability among studies (Benjamin et al., 2021). This broad range suggests potential fluctuations in how metabolic fatigue influenced performance, possibly affected by different players’ conditions or experimental settings. Metabolic fatigue primarily arises from the depletion of glycogen stores, accumulation of metabolic by-products like lactate, and disturbances in ion homeostasis. Soccer is characterized by intermittent high-intensity efforts, including sprints, jumps, and rapid changes of direction, interspersed with periods of moderate and low-intensity activity. These activities rely predominantly on aerobic and anaerobic energy systems, which leads to substantial metabolic demands. The accumulation of blood lactate and hydrogen ions during high-intensity efforts contributes to metabolic acidosis, impairing muscle contractility, and overall performance (Bangsbo et al., 2006; Krustrup et al., 2006). Rampinini et al. (2007a, 2007b) highlighted the relationship between muscle glycogen depletion and reduced high intensity running capacity toward the latter stages of a match. Furthermore, the balance of ion gradients, particularly calcium and potassium, was disrupted during prolonged exercise, contributing to muscle fatigue and reduced neuromuscular function (Mohr et al., 2005; Skalski et al., 2024).

Increased production of reactive oxygen species (ROS) during extensive exercise can lead to oxidative damage, further exacerbating metabolic fatigue. Effective strategies to mitigate metabolic fatigue include nutritional interventions, such as carbohydrate loading and in-game carbohydrate supplementation, which have been shown to delay glycogen depletion and improve performance (Cermak and van Loon, 2013). Furthermore, conditioning programmes that focus on both aerobic and anaerobic capacity can improve energy production and utilisation efficiency, thus reducing the appearance of metabolic fatigue (Iaia et al., 2009). In this study, the high variability and range of metabolic fatigue could be due to diverse physiological responses influenced by various metabolic conditions or interventions (Abbott et al., 2023). The negative minimum effect size could be interesting to explore further, as it might indicate conditions under which metabolic efficiency was reduced. Several nutritional supplements, such as betaine, caffeine, curcumin, ketone ester, and protein, have been evaluated, with generally positive results. Betaine supplementation was remarkable with a notable mean effect size of 0.74 (Benjamin et al., 2021). The analysis of metabolic fatigue revealed an I^2^ value of 98.73%, indicating very high heterogeneity. This level of variability suggests that while there were consistent trends in how metabolic fatigue impacted soccer performance, there were also significant influences from variables that differed across studies, such as the intensity of exercise or individual metabolic differences among players.

### Neuromuscular Fatigue

Neuromuscular fatigue in soccer emerged with a notably high mean effect size of 0.48, underscoring a substantial and consistent impact on performance. This type of fatigue involves the failure of the nervous system to sustain the force output required for high-performance activities and is influenced by both central (neural) and peripheral (muscular) factors (Thorpe et al., 2017; Skalski et al., 2025). Interventions such as neuromuscular and plyometric training have shown robust effectiveness, with mean effect sizes exceeding 1.2 (Wang and Zhang, 2016). Neuromuscular fatigue, with an I^2^ of 99.79%, exhibits extremely high heterogeneity among the studies analyzed, suggesting that the effects of neuromuscular fatigue on soccer performance were relatively uniform across different research settings and methodologies. This makes neuromuscular fatigue a particularly reliable target for interventions designed to optimize performance, as strategies developed in this area are likely to be effective across diverse players’ populations and competitive environments. Rampinini et al. (2008) demonstrated that the accumulation of fatigue during a match could drastically reduce sprinting speed, jumping ability, and overall agility, which are all crucial for high-level performance in soccer. Ispirlidis et al. (2008) found that intermittent sprinting and rapid direction changes were primary contributors to muscle damage, leading to neuromuscular fatigue. Furthermore, Mohr et al. (2005) revealed that a decline in muscle function related to fatigue was not only due to metabolic changes, but also to alterations in the neural aspects of muscle contraction, suggesting that physical and neurological factors need to be addressed in recovery strategies.

### Psychological Fatigue

Psychological fatigue significantly affected soccer performance, with a mean effect size of 0.55, which is substantial but less consistent, as indicated by a wide confidence interval ranging from 0.21 to 0.89. This variability suggests that the impact of psychological factors on performance might be highly context-dependent, varying greatly among players or situational conditions. The standard deviation of effect size was highest for psychological fatigue (0.17), suggesting considerable variability in the results within this category (Smith et al., 2020). Psychological fatigue also presented a more complex picture with an I^2^ of 97.08%, falling into the category of very high heterogeneity. Psychological fatigue can be described as a decrease in mental performance resulting from prolonged periods of cognitive activity or emotional stress, leading to decreased motivation, concentration, and decision-making abilities. This type of fatigue often interacts with physical fatigue, exacerbating its effects and vice versa. Kruk et al. (2001) observed that caffeine ingestion could improve psychomotor performance during exercise, suggesting that interventions targeting arousal levels could mitigate some aspects of psychological fatigue in athletes. Additionally, Segers et al. (2023) highlighted the importance of monitoring and managing training loads to prevent excessive fatigue and maintain optimal performance levels throughout a competitive season. Research has consistently confirmed that psychological fatigue affects athletes' performance, with diverse impact based on the severity and the context of fatigue (Mendes et al., 2022). Cognitive functions like decision-making and concentration are significantly impaired by psychological fatigue, influencing both individual performance and team dynamics. Psychological fatigue can lead to decreased reaction time and poor judgment, which are critical in high-speed strategic game situations such as in soccer.

### Multimodal Fatigue and Psychomotor Fatigue Threshold

Our findings align with previous research, suggesting a generalized moderate impact of fatigue, but also highlighting the variability in how fatigue manifests across different studies and soccer-related activities. This study also indicates the necessity of a multimodal model that can guide targeted interventions, such as specific training regimens (HIIT, neuromuscular training) and recovery strategies (nutrition, physiotherapy), to address the multifaceted aspects of fatigue that affect players’ performance differentially. The concept of the psychomotor fatigue threshold integrates these findings, illustrating how different types of fatigue are interrelated.

Beyond the well-established physical, neuromuscular, metabolic, and psychological effects, psychomotor fatigue plays a critical role in compromising technical execution and tactical responsiveness in soccer (Chmura and Nazar, 2010). As fatigue accumulates during training and/or competition, athletes exhibit diminished decision-making speed, reduced spatial and situational awareness, and impaired tactical adaptability. These changes manifest as delays in transitions, mistimed pressing efforts, positional disorganization, and breakdowns in coordinated team behavior—especially during high-stress match periods, such as the final 15 minutes.

From the technical side, fatigue-related performance decline is consistently associated with decreased passing accuracy, reduced ball control, lower dribbling success, and diminished shooting precision (Barte et al., 2019; Mendes et al., 2022). Such impairment can disrupt not only individual execution, but also the broader tactical dynamics of the team. The compounded effects of late-game muscular fatigue and cognitive overloading are linked to an increased incidence of non-contact injuries, particularly muscle strains and neuromechanical failures (Chmura et al., 2019; Pereira et al., 2022).

From a coaching perspective, mitigating fatigue requires both strategic rotation of players and tactical system flexibility. Adjustments such as staggered pressing, zonal compactness, or formation changes enable teams to reduce cognitive and physical demands without compromising competitive intensity. Similarly, training microcycles can be optimized by alternating high-cognitive-load sessions with low-load recovery-oriented drills, thus balancing adaptation with the avoidance of overtraining syndromes. Understanding its threshold can help in optimizing players’ output and reducing the risk of performance decline during critical game phases. As soccer is a high-intensity sport requiring constant decision making and tactical awareness, psychomotor fatigue can affect these cognitive functions, leading to decreased performance and increased risk of injury (Moreira et al., 2025). Identifying a threshold for psychomotor fatigue could help better manage these risks. Soccer players vary in their response to fatigue due to individual differences in physiology, psychology, and fitness levels. Understanding and establishing thresholds for psychomotor fatigue would allow for more personalised monitoring and management strategies, enhancing both safety and performance.

### Recovery Strategies

Effective fatigue management needs to integrate both physiological recovery and cognitive restoration (de Dios-Álvarez et al., 2024). Evidence supports structured interventions including nutritional periodization (carbohydrate-protein intake), hydration protocols, targeted sleep hygiene, and modalities such as cold-water immersion, massage, and active recovery as means of accelerating muscle recovery and systemic recalibration (Nédélec et al., 2012a, 2012b; Pinheiro et al., 2024; Skalski et al., 2025).

From the mental performance side, meditative techniques, motor imagery, and neurofeedback training have demonstrated efficacy in regulating psychophysiological stress responses and prolonging attentional control under fatigue. These methods can help athletes sustain tactical decision-making quality and emotional regulation, especially in pressure-intense scenarios (Van Cutsem and Marcora, 2021).

### Research Gaps and Future Directions

The current study primarily focused on male soccer players. Future studies could expand to include women and youth players to understand whether there are differences in fatigue impact and management between genders and age groups. It also focussed on studies within certain regions or levels of play. Expanding the geographical scope could provide insights into how environmental and cultural factors affect fatigue and performance. Since most of the studies were cross-sectional, longitudinal studies could help understand the long-term effects of fatigue and the effectiveness of different management strategies over time. Future investigations should prioritize the development of AI-assisted diagnostic tools, wearable biosensors, and augmented reality environments for the real-time monitoring of psychomotor function during training and competition. These tools offer the potential to detect fatigue risk early and tailor interventions based on multimodal data streams (Gallo et al., 2017; Pereira et al., 2022).

## Limitations

This study faced several limitations that should be considered when interpreting the findings. Most of the included studies focused on male soccer players, which limits the generalizability of the findings to female players and other sports disciplines. There was significant heterogeneity in the tools and methods used to measure and report fatigue across studies, which could influence the consistency and comparability of the results. While the study examined multiple dimensions of fatigue, the interrelationships among different types of fatigue and their cumulative impact on performance and recovery were not fully explored. In addition, there was a marked variability in the impact of psychological fatigue on performance. Although minimal publication bias was detected, the possibility of unpublished negative findings cannot be completely ruled out, potentially skewing the overall understanding of fatigue impact. The varying conditions under which the studies were conducted, such as environmental factors and players’ nutrition, were not uniformly controlled, possibly affecting the outcomes.

## Conclusions

This study reinstates the need to derive a multimodal model to represent fatigue in soccer, which is underscored by the complex and dynamic nature of the sport. Understanding psychomotor fatigue and establishing a threshold for it is crucial to optimise players’ performance, prevent injuries, and extend players' careers. This systematic review and meta-analysis explored the multifactorial thresholds of psychomotor fatigue in soccer, examining how various types of fatigue—physical, psychological, neuromuscular, and metabolic—affect players’ performance and injury risk. The study highlights the significant impact of neuromuscular fatigue on performance, consistent across different players’ performance levels, and the variable impact of psychological and metabolic fatigue influenced by contextual factors such as match conditions and individual players’ characteristics. Physical fatigue, although less variable, similarly affects players’ output and recovery. The research underscores the importance of comprehensive fatigue management strategies, including innovative recovery techniques and training modifications, to enhance players’ performance and career longevity.

## Practical Implications

The practical implications of the study on multifactorial thresholds of psychomotor fatigue in soccer are essential to improve players’ performance, prolong career, and reduce injury risks. Here are the concise practical implications:

- Determine the psychomotor fatigue threshold for players under different conditions, and utilize insights from the distinct fatigue types—physical, psychological, neuromuscular, and metabolic—to develop customized training plans.
- Integrate effective recovery methods highlighted in the study, such as nutritional supplements (betaine, caffeine, and curcumin) and training adjustments, to accelerate recovery and enhance performance.
- Apply wearable technology and monitoring tools like GPS and heart rate monitors to track fatigue in real-time, enabling personalized adjustments to training and recovery plans.
- Incorporate mental resilience and cognitive training into regular routines to help athletes manage stress and maintain sharp decision-making even under fatigue.
- Recognize and accommodate individual variability in fatigue response, particularly between younger and more experienced players, to create customized management strategies.
- Implement comprehensive fatigue management to potentially extend players’ careers by minimizing cumulative physical strain and enhancing long-term health and performance.
